# Invasive pulmonary aspergillosis evaluation in hematology patients: Three years results of tertiary hospital

**DOI:** 10.17305/bb.2024.10766

**Published:** 2024-07-17

**Authors:** Mine Aydın Kurc, Betül Günaydın, Seval Akpınar, Birol Safak, Nuri Kiraz

**Affiliations:** 1 Department of Medical Microbiology, Tekirdag Namik Kemal University, Tekirdag, Türkiye; 2 Microbiology Laboratory, Uşak Training and Research Hospital, Usak, Türkiye; 3 Department of Internal Diseases, Tekirdag Namik Kemal University, Tekirdag, Türkiye; 4 Department of Medical Microbiology, Atlas University, Istanbul, Türkiye; 5 Department of Medical Microbiology, Istanbul University-Cerrahpașa, Istanbul, Türkiye

**Keywords:** Invasive pulmonary aspergillosis (IPA), galactomannan testing, hematological malignancy

## Abstract

Invasive pulmonary aspergillosis (IPA) is the most frequent invasive fungal disease occurring in patients with hematological malignancies. Serum galactomannan (GM) antigen monitoring is thought to be helpful in the diagnosis of IPA. The aim of this study was to determine the role of a GM assay in serum samples for the diagnosis of IPA in patients with hematological disease. The data of 366 immunosuppressed patients who were hospitalized and followed up in the hematology clinic from January 2017 to December 2019 were retrospectively analyzed. The clinical and radiological findings of the patients and the GM results, requested twice a week, were evaluated. In this study, the incidence of probable and possible IPA was determined to be 15.3% (56/366). Of the cases detected, 28 (50.0%) were patients diagnosed with acute myeloid leukemia (AML), and 34 (60.7%) patients who had compatible clinical and examination findings were started on antifungal treatment. Additionally, area under the curve (AUC) values were calculated by receiver operating characteristic (ROC) analysis, and it was determined that the diagnostic efficiency was more predictive when the cut-off was 0.5 in the GM test for IPA disease. The detection of GM antigen in serum is a very useful and rapid method for diagnosing IPA disease in immunosuppressed hematology patients. However, GM results should be evaluated together with clinical and radiological findings for early diagnosis, and the treatment approach should be determined accordingly.

## Introduction

*Aspergillus* is defined as a fungus belonging to the Ascomycota phylum, characterized by multicellular, branched structures called hyphae. *Aspergillus* species proliferate in nitrogen and carbon sources and are thermotolerant (proliferates at 37 ^∘^C–50 ^∘^C) due to their ribosomal proteins. They are also resistant to high osmotic pressures [1]. This filamentous fungus is commonly found in the environment and in areas where health services are provided. It spreads to susceptible individuals through its airborne conidia. Although conidia are inhaled daily, most people do not develop *Aspergillus*-related diseases due to the normal human immune system’s defenses, which include alveolar macrophages, neutrophils, monocytes, and natural killer cells [2].

*Aspergillus* species are implicated in various clinical conditions. These include invasive aspergillosis and superficial infections (such as otomycosis, keratitis, and burn wound infections), which affect more than 300,000 individuals annually. Additionally, allergic bronchopulmonary disease and rhinosinusitis impact over ten million individuals globally, while chronic pulmonary and rhinosinusital aspergillosis affect approximately three million individuals [3].

Rapid diagnosis is crucial in invasive pulmonary aspergillosis (IPA) due to the high mortality and morbidity rates, especially in severely immunosuppressed patients with limited host defenses, such as those who have undergone solid organ transplantation, have hematological malignancies, or suffer from neutropenia [2, 4]. However, diagnosis is challenging due to nonspecific clinical-radiological findings, the need for invasive sampling, and the low sensitivity of traditional culturing and histopathological methods [5]. The galactomannan antigen (GM) test offers a potential noninvasive diagnostic method. However, certain antifungals and antibiotics can affect the accuracy of the GM test, occasionally resulting in false-positive or false-negative results [4].

According to the guidelines of the Infectious Diseases Society of America, serum and bronchoalveolar lavage (BAL) GM detection provide high-quality evidence in diagnosing invasive aspergillosis in adult and pediatric patients with hematological malignancies or undergoing hematopoietic stem cell transplantation, and are strongly recommended as correct markers. While routine serum GM screening is not advised for patients taking antifungal treatment or prophylaxis, the GM test is recommended for bronchoscopic materials [6].

However, the combined application of a weekly GM test and computerized tomography (CT) is suggested to be effective in patients receiving antifungal treatment [4]. Conversely, GM is not recommended for screening in solid organ recipients and those suffering from chronic granulomatous disease. Additionally, the 1,3-Beta-D-Glucan test is advised for diagnosing invasive aspergillosis, although it is not specific to *Aspergillus* [6]. GM sensitivity is considerably lower in non-neutropenic patients compared to neutropenic patients. GM optical density index (ODI) levels are a reliable indicator for determining the success of antifungal treatments [7].

This study aimed to investigate and compare the diagnostic value of the serum GM antigen test in immunosuppressed patients hospitalized in our hematology clinic and suspected of having IPA, as well as to determine the optimal GM cutoff value in our patient group.

## Materials and methods

### Patient group

The data from hospitalized patients who were treated and followed up in the hematology clinic of Tekirdağ Namık Kemal University Hospital between 2017 and 2019 were retrospectively analyzed in our study. The patients’ clinical and radiological findings, as well as the GM results requested twice weekly, were evaluated.

Demographic data, radiological and clinical findings, extended antibiotic use, risk factors, such as neutropenia, histopathology, and fungal biomarkers of the patients were analyzed. Additionally, the total number of GM serum tests and the number of positive and negative results were recorded. Antifungal treatments administered for IPA despite negative test results were also noted.

Radiological findings, including pulmonary infiltrations, consolidation, halo sign, cavity or air-crescent sign, cavitation, lesions with well-defined boundaries, and dense nodules, were evaluated for invasive aspergillosis in high-resolution lung CT scans of all patients. Based on this data, the occurrence of clinical findings in patients with suspicious lung lesions was assessed.

### GM testing

The GM test was performed on serum samples collected from patients at least twice a week using the Sandwich-ELISA method and the Platelia Aspergillus Ag kit (Platelia™ Aspergillus, Bio-Rad, USA), following the manufacturer’s instructions. The ODI value of the samples was measured using a microplate reader, and the GM test results in the serum samples were calculated. GM test results with an ODI value of 0.5 and above were regarded as positive.

### IPA diagnostic criteria

The European Organization for Research and Treatment of Cancer/Mycoses Study Group (EORTC/MSG) 2008 criteria were used to diagnose patients with IPA [8]. Patients were categorized as having no IPA, proven IPA, probable IPA, or possible IPA.

According to these criteria: (i) *Proven IPA:* Presence of hyphae in histopathological and direct microscopic examinations, and presence of *Aspergillus sp.* growth in culture. (ii) *Probable IPA:* Presence of at least one host factor (neutropenia, solid organ transplant, connective tissue disorders, or use of immunosuppressive agents such as corticosteroids), at least one clinical criterion (halo sign, air-crescent sign, or cavity), and mycological criteria (such as a positive *Aspergillus sp.* culture from qualified specimens or a positive serum GM detection result at a cutoff value of ≥ 0.5). (iii) *Possible IPA:* Presence of at least one host factor and one clinical criterion, but absence of mycological criteria.

### Ethical statement

This study was approved by the Non-Interventional Clinical Research Ethics Committee of Tekirdağ Namık Kemal University (approval number: 2019/111/07/07, dated 27-06-2019). We confirm that the study was conducted in accordance with the relevant guidelines and regulations.

### Statistical analysis

The results obtained in the study were statistically analyzed using the SPSS 20.0 (SPSS Inc., Chicago, IL, USA) software package program. Descriptive statistics are presented as mean ± standard deviation for numeric variables and as number (*n*) and percentage (%) for categorical variables. The non-parametric Spearman’s correlation test was applied to evaluate the relationship between data. The Kruskal–Wallis test was used to detect differences between quantitative variables of patients diagnosed and not diagnosed with IPA regarding the GM test, and the Mann–Whitney *U* test was used to detect differences between the two groups. Study data were evaluated at a 95% confidence interval and two-tailed. A value of *P* < 0.05 was accepted as statistically significant. Finally, a receiver operating characteristic (ROC) curve was constructed to determine the optimum cut-off value for the patient group’s GM test.

## Results

### Patient characteristics

Patients who received treatment and follow-up at our hospital’s Hematology Clinic between 2017 and 2019 are the focus of our study. A total of 366 patients, aged 16–90 years (58.57 ± 16.85), were included. Among these patients, 174 (47.5%) were female and 192 (52.5%) were male. The most common underlying diseases were acute myeloid leukemia (AML) in 79 cases (21.6%), multiple myeloma (MM) in 59 cases (16.1%), non-Hodgkin lymphoma (NHL) in 51 cases (13.9%)—of which 60.8% were aggressive lymphoma and 39.2% were indolent lymphoma—and chronic lymphocytic leukemia (CLL) in 32 cases (8.7%). Other hematological diseases, such as hemophagocytic syndrome, undiagnosed leukocytosis and leukopenia, idiopathic thrombocytopenic purpura, polycythemia vera, and myelodysplastic syndrome, accounted for 67 cases (18.3%), while various anemias, including iron deficiency anemia, aplastic anemia, hemolytic anemia, and megaloblastic anemia, were present in 33 cases (9.0%) (Table 1).

**Table 1:** Demographic characteristics of the patients

**Characteristics**	***n* (%)**
Men	192 (52.5%)
Women	174 (47.5%)
Age (mean), years (range)	58.57 ± 16.85 (16–90)
*Diagnosis*	
AML	79 (21.6%)
MM	59 (16.1%)
NHL	51 (13.9%)
CLL	32 (8.7%)
ALL	18 (4.9%)
HL	8 (2.2%)
CML	7 (1.9%)
HCL	6 (1.6%)
Solid organ malignancy	6 (1.6%)
Other hematological disease	67 (18.3%)
Anemia	33 (9.0%)

AML: Acute myeloid leukemia; MM: Multiple myeloma; NHL: Non-hodgkin lymphoma; CLL: Chronic lymphocytic leukemia; ALL: Acute lymphocytic leukemia; HL: Hodgkin’s lymphoma; CML: Chronic myeloid leukemia; HCL: Hairy cell leukemia; Solid organ malignancy: such as lung and gynecological cancers, Ewig’s sarcoma, Metastatic tumor; Other hematological disease: Hemophagocytic syndrome, Undiagnosed cause unknown leukocytosis and leukopenia, Idiopathic thrombocytopenic purpura, Polycythemia vera, Myelodysplastic syndrome; Anemia: Iron deficiency anemia, Anemia, Aplastic anemia, Hemolytic anemia, Megaloblastic anemia.

An abnormal finding was identified in high-resolution CT scans in 94 cases (25.7%). Among the 128 patients with positive GM results, 57 (44.5%) had these abnormal CT findings.

### GM testing results and antifungal treatment

A total of 2053 GM tests were conducted at different times on the patient group (*n* ═ 366). GM-positive results were detected in 128 (35.1%) of the patients. Recurrent positive results were identified in 45 (35.1%) of these 128 patients. Radiological evidence was found in 57 (44.5%) of the GM-positive patients, while 79 (61.7%) had neutropenia.

In our study, when the GM test results of our patient group were evaluated, the most common underlying disease was AML (*n* ═ 79). Of these patients, 40 had a positive GM test and 33 had neutropenia. Additionally, the GM test was negative in 39 patients, and neutropenia was found in 25 of these patients.

Antifungal treatment was initiated in 53 (41.4%) of patients with positive serum GM tests. Among these, 38 (29.7%) received posaconazole, 7 (5.4%) received voriconazole, 6 (4.7%) received fluconazole, and 2 (1.6%) received anidulafungin treatment, while 3 received antifungal agents for different reasons. Additionally, antifungal treatment was initiated by clinicians in 23 patients despite their GM antigen test result being negative (*n* ═ 238) (Table 2).

**Table 2:** IPA diagnoses, GM test results, CT, neutropenia, and antifungal treatment findings of patients

**Classification of fungal infection**	**No. of patients**	**GM testing**		**CT finding**	**Neutropenia**	**Antifungal treatment**
		**Positive**	**Negative**			
IPA cases	56 (15.3%)	44 (78.6%)	12 (21.4%)	56 (100%)	56 (100%)	34 (60.7%)
Non IPA cases	310 (84.7%)	84 (27.1%)	226 (72.9%)	38 (12.2%)	90 (29.0%)	42 (13.5%)
All patients	366 (100%)	128 (35%)	238 (65%)	94 (25.7%)	146 (39.9%)	76 (20.8%)

IPA: Invasive pulmonary aspergillosis; GM: Galactomannan antigen; CT: Computerized tomography.

In our study, patients with positive and negative GM test results were compared from various aspects. A significant relationship was found between GM test positivity and both the initiation of antifungal treatment and the presence of abnormal findings on CT scans (*P* < 0.001).

### Patients diagnosed with IPA

Since biopsy or needle aspiration biopsy samples were not obtained for histopathological and culture examination in any of the patients, a definitive diagnosis of IPA could not be established according to EORTC/MSG criteria.

**Figure 1.**
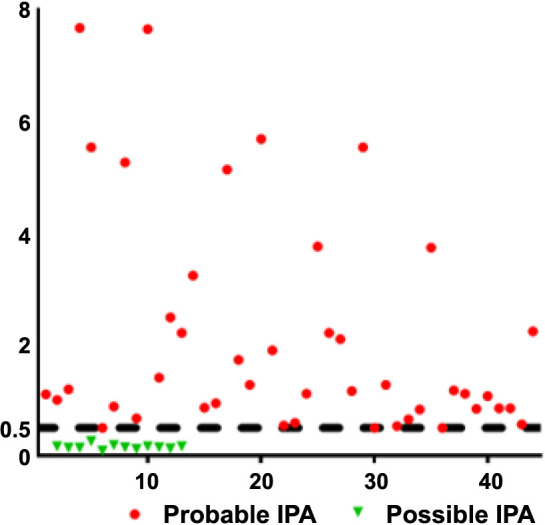
**Scatter diagram displaying the GM ODI value of patients diagnosed with IPA.** IPA: Invasive pulmonary aspergillosis; GM: Galactomannan antigen; ODI: Optical density index.

The number of patients diagnosed with probable IPA was 44 (12.0%), and those with possible IPA were 12 (3.3%). The 56 (15.3%) patients who received a probable or possible IPA diagnosis had ages ranging between 19 and 88 years (57.03 ± 15.4). Among these, 39 (69.6%) were male, and 28 (50.0%) were diagnosed with AML. Among them, 34 (60.7%) patients with consistent clinical and examination findings were diagnosed with IPA, and antifungal treatment was initiated. However, antifungal treatment was started in 76 (20.8%) of the patients regardless of an IPA diagnosis. In our study, 310 (84.7%) patients did not receive a diagnosis of IPA according to the EORTC/MSG criteria (Figure 1 and Table 2).

In this study, GM-positive results were detected in 128 patients. Among these patients, 44 were diagnosed with IPA, while 84 were not diagnosed with IPA. When the GM ODI value was evaluated, the proportion of patients with an ODI value ≥ 0.5 was high in both groups (Table 3). Additionally, recurrent GM test positivity was detected in 45 patients diagnosed with IPA and in 24 patients in the other group.

**Table 3:** Distribution of GM ODI values of patients with and without a diagnosis of IPA

**Variable**	**IPA cases (*n* ═ 56)**	**Non IPA cases (*n* ═ 310)**
GM positive results	44 (100%)	84 (100%)
ODI ≥ 0.5	16 (36.4%)	45 (53.6%)
ODI ≥ 1.0	11 (25.0%)	14 (16.7%)
ODI ≥ 1.5	2 (4.5%)	6 (7.1%)
ODI ≥ 2.0	15 (34.1%)	19 (22.6%)

IPA: Invasive pulmonary aspergillosis; GM: Galactomannan antigen; ODI: Optical density index.

The mortality rate in patients diagnosed with IPA according to EORTC/MSG criteria was determined to be 25% (*n* ═ 14), compared to 6.12% (*n* ═ 19) in patients not diagnosed with IPA.

### Detection of optimal cut-off value effect for GM testing

**Figure 2.**
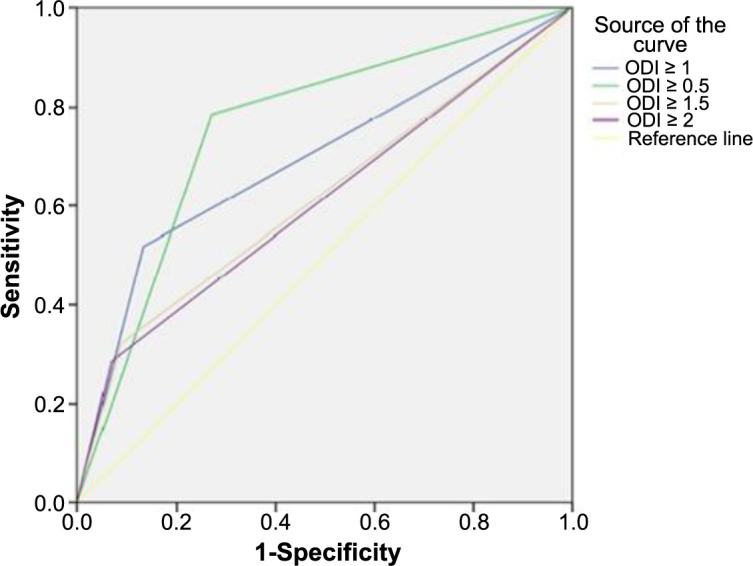
**ROC curve for serum GM detection in patients with IPA.** IPA: Invasive pulmonary aspergillosis; GM: Galactomannan antigen; ROC: Receiver operating characteristic; ODI: Optical density index.

In our study, the ROC curve was constructed according to different GM cut-off values (ODI value: 0.5, 1.0, 1.5, 2.0), and the area under the curve (AUC) values were calculated for diagnostic efficiency in IPA disease. These values were determined to be most decisive in terms of diagnostic efficiency at a cut-off of 0.5 (AUC: 0.757; standard error: 0.035; 95% confidence interval: 0.689–0.826). However, it was found that sensitivity and specificity were highest at the accepted value, and as the cut-off value increased, sensitivity decreased (Figure 2).

## Discussion

IPA is considered a disease of immunocompromised patients [2]. However, clinical and radiological findings are also not specific. The challenges in diagnosis arise from the requirement of invasive procedures for a definitive diagnosis and the frequent presence of thrombocytopenia and coagulation disorders in patients. Fungal culture, on the other hand, is time consuming and has low positivity rates. Therefore, non-invasive and rapid diagnostic methods are used [4, 5, 9]. GM, Beta-D glucan, PCR, *Aspergillus* lateral flow, urinary antigen, siderophores, cytokines, and pentraxin-3 detection, PET/CT, and immuno PET/MRI are among these methods [10–17]. The use of different diagnostic methods simultaneously increases the accuracy of the diagnosis [10, 18]. Among these, GM is a cell wall polysaccharide of *Aspergillus* species and, although it proliferates in invasive infections, it can be detected in serum and other body fluids. It can be detected in the circulation 5–8 days before clinical symptoms appear. Additionally, two consecutive test results with an ODI value ≥0.5 are required for the highest test accuracy [19]. Moreover, two serum GM tests must be carried out weekly to initiate preemptive treatment that increases survival [18]. IPA is seen more frequently in AML patients compared to other patient groups [4, 18, 20]. However, risk factors, such as diabetes mellitus, severe influenza disease, and chronic granulomatous disease are also reported [18]. Invasive diagnostic methods are not preferred for patient monitoring in our hospital’s hematology clinic due to the risk of complications. Among noninvasive tests, GM is used twice weekly. GM tests were conducted on 366 patients hospitalized in our clinic with various hematological diagnoses, and AML patients accounted for 21.6% of them. Positive results were detected in 128 (35.1%) of the patients, and 45 (35.1%) of these had recurrent positive results. Among patients diagnosed with IPA and initiated on antifungal treatment (*n* ═ 26), recurrent positivity was detected in 80.8% (*n* ═ 21) of cases.

While the diagnosis of IPA is increasingly recognized, many cases go unnoticed, and diagnosis can only be made post-mortem [18, 21]. The EB-A2 monoclonal antibody is used to detect the β-1,5 galactopyranosyl antigenic side chain in the GM ELISA test [22]. In high-risk patients, false negativity in the GM test can occur due to the use of mold-active antifungal prophylaxis [10, 20]. Additionally, sensitivity is low in non-neutropenic patients [7, 19]. False positives may be seen due to reasons such as beta-lactam antibiotic use (piperacillin-tazobactam, amoxicillin-clavulanate), transfusion, Plasmalyt infusion, histoplasmosis, fusariosis, *Bifidobacterium spp*., MM, severe mucositis, IV immunoglobulin, and *Aspergillus*-contaminated foods [10, 19, 22]. The rate of false-positive results was determined to be 14% in a study that emphasized the importance of considering fungi other than *Aspergillus* as well [23]. Similarly, false-positive results were detected in 21 (5.7%) patients not diagnosed with IPA in our study. Despite numerous factors that may influence the results, strong recommendation and high-quality evidence suggest the use of BAL and serum GM measurements [6].

Host factors and clinical criteria compliant with EORTC/MSG standards are used in addition to GM testing [8]. Twice-weekly GM screening in neutropenic patients, combined with clinical and radiological findings, is currently considered the most appropriate approach for diagnosis [6]. The sensitivity of serial GM screening is quite high in neutropenic patients [7]. Weekly GM measurements and CT monitoring are effective in the early diagnosis of IPA, even in febrile neutropenic patients receiving antifungal treatment [4]. In our study, 44 (12.0%) patients with GM-positive results, a neutrophil count <500/mm^3^, and positive CT findings were diagnosed with probable IPA, while 12 (3.3%) patients with GM-negative results, a neutrophil count <500/mm^3^, and positive CT findings were diagnosed with IPA. The percentage of patients diagnosed with IPA in different studies has been reported as 6.1%–13.4% [24–27]. In our study, 15.3% of patients undergoing follow-up for GM were diagnosed with IPA. Our study results show that GM positivity is consistent with radiological findings in patients considered to have IPA. In another study comparing the presence of radiological findings with GM positivity, it was determined that GM positivity was highly consistent with radiological findings [28]. These data suggest that GM positivity before the detection of radiological findings may be helpful in diagnosing IPA.

Although studies on *Aspergillus* species indicate that the rate of resistance to some antifungals (azole resistance) varies [29–32], a separate study reported no resistance to voriconazole and amphotericin B [31]. Resistance rates differ according to the *Aspergillus* species. In one study, Amphotericin B resistance rates were reported as 11.8% for *A. fumigatus*, 10% for *A. flavus*, and 33.3% for *A. niger*; Itraconazole resistance was 11.8% for *A. fumigatus*, 20% for *A. flavus*, and 33.3% for *A. niger*, while no resistance to caspofungin was observed. The mortality rate in aspergillosis has been reported as 26.7% [33]. According to another study, the mortality rate was reported as 29.2% in the group treated with voriconazole and 42.1% in the group treated with amphotericin B [34]. In our study, among the 56 patients diagnosed with probable or possible IPA according to the EORTC/MSG criteria, the mortality rate was determined as 25% (*n* ═ 14) in patients diagnosed with IPA based on clinical findings and initiated treatment.

It has been reported that the rate of GM decrease in response to antifungal treatment is important for assessing mortality [6, 7, 10]. Patients who responded successfully to voriconazole treatment showed earlier decreases in GM values compared to those whose response failed at the end of treatment [6]. The high mortality rate (55.5%) observed in patients receiving voriconazole (*n* ═ 9) at our hospital is particularly noteworthy. GM levels should be monitored during treatment follow-up to reduce the mortality rate. If the expected reduction is not observed, highly azole-resistant non-fumigatus species should be considered as probable causative agents of IPA.

Clinically significant ODI cut-off determination is essential in terms of clinical application for patients with hematologic malignancies due to their high mortality rates. One important factor influencing the test’s sensitivity and specificity is the choice of cutoff point for positivity, which determines whether or not results qualify as true positives. In our study, the generated ROC curve confirmed that the test had good performance, even at low cutoffs. Similar to previous studies, high overall sensitivity and acceptable specificity were obtained by using GM ODI value at a cutoff of 0.5 to define an IPA case. The most significant limitation of our study is the lack of species identification and sensitivity testing in Aspergillosis due to the inability to perform invasive procedures. However, high mortality rates in patients receiving voriconazole treatment suggest that voriconazole resistance may be high and that non-fumigatus *Aspergillus* species may also be involved.

## Conclusion

In conclusion, patients can be monitored using CT findings and noninvasive, rapid GM testing, due to the difficulty of implementing invasive procedures required for a definitive diagnosis. These procedures are associated with the risk of complications, and fungal culturing is time consuming and has a low positivity rate. Additionally, local epidemiological data should be continuously monitored to reduce mortality, and treatment approaches should be determined accordingly.
